# Wearable Movement Exploration Device with Machine Learning Algorithm for Screening and Tracking Diabetic Neuropathy—A Cross-Sectional, Diagnostic, Comparative Study

**DOI:** 10.3390/bios14040166

**Published:** 2024-03-29

**Authors:** Goran Radunovic, Zoran Velickovic, Slavica Pavlov-Dolijanovic, Sasa Janjic, Biljana Stojic, Irena Jeftovic Velkova, Nikola Suljagic, Ivan Soldatovic

**Affiliations:** 1 Institute of Rheumatology, 11000 Belgrade, Serbia; velickovic.z@yahoo.com (Z.V.); slavicapavlovdolijanovic@gmail.com (S.P.-D.); sasajanjic08@gmail.com (S.J.); b.stojic@yahoo.com (B.S.); 2 Faculty of Medicine, University of Belgrade, 11000 Belgrade, Serbia; 3 DIVS Neuroinformatics DOO, 11000 Belgrade, Serbia; irenajv@divstechnology.com (I.J.V.); nsuljagic@divstechnology.com (N.S.); 4 General Hospital Loznica, 15300 Loznica, Serbia; 5 Faculty of Electrical Engineering, University of Belgrade, 11000 Belgrade, Serbia

**Keywords:** wearable device, diabetic neuropathy, screening, tracking

## Abstract

Background: Diabetic neuropathy is one of the most common complications of diabetes mellitus. The aim of this study is to evaluate the Moveo device, a novel device that uses a machine learning (ML) algorithm to detect and track diabetic neuropathy. The Moveo device comprises 4 sensors positioned on the back of the hands and feet accompanied by a mobile application that gathers data and ML algorithms that are hosted on a cloud platform. The sensors measure movement signals, which are then transferred to the cloud through the mobile application. The cloud triggers a pipeline for feature extraction and subsequently feeds the ML model with these extracted features. Methods: The pilot study included 23 participants. Eleven patients with diabetes and suspected diabetic neuropathy were included in the experimental group. In the control group, 8 patients had suspected radiculopathy, and 4 participants were healthy. All participants underwent an electrodiagnostic examination (EDx) and a Moveo examination, which consists of sensors placed on the feet and back of the participant’s hands and use of the mobile application. The participant performs six tests that are part of a standard neurological examination, and a ML algorithm calculates the probability of diabetic neuropathy. A user experience questionnaire was used to compare participant experiences with regard to both methods. Results: The total accuracy of the algorithm is 82.1%, with 78% sensitivity and 87% specificity. A high linear correlation up to 0.722 was observed between Moveo and EDx features, which underpins the model’s adequacy. The user experience questionnaire revealed that the majority of patients preferred the less painful method. Conclusions: Moveo represents an accurate, easy-to-use device suitable for home environments, showing promising results and potential for future usage.

## 1. Introduction

The prevalence of diabetes mellitus has seen a marked increase during the second half of the 20th century, with a significant escalation at the beginning of the 21st century. According to data from the USA, the prevalence has even doubled [1], regardless of gender, age, or socioeconomic status [2]. In the last decade, a stabilization of prevalence and incidence has been first observed in countries with a high standard of living. The aforementioned trends are often viewed as a reflection of improved living standards, characterized by longer life expectancies and reduced mortality rates [2,3,4,5].

Chronic hyperglycemia, especially when the disease is not adequately managed, can lead to a more frequent occurrence of chronic microvascular damage to sensitive organs and tissues. This includes damage to the peripheral nerve fibers, clinically manifesting as diabetic peripheral neuropathy, a prevalent and debilitating condition [6,7]. Distal symmetric polyneuropathy (DSP) represents the most common form of diabetic peripheral neuropathy; however, various patterns of nerve injury can also occur [8]. Peripheral neuropathy is the most common chronic complication of diabetes, developing in almost half of patients [9,10,11]. The condition consequentially leads to severe morbidities, such as neuropathic pain, skin ulcers [11,12,13], and even amputation. Beyond the direct health impacts, the complexity of diagnosing and treating diabetic neuropathy significantly escalates its economic costs, burdening healthcare systems and society [8,10].

The prevalence of peripheral neuropathy among adults with diabetes is estimated to range from 6% to 51% and is influenced by factors such as age, diabetes duration, glycemic control, type of diabetes, and several other factors. Through the improvement of early diagnostics and new therapeutic modalities, the life expectancy of patients suffering from this disease is expected to increase significantly [3]. Consequently, an increase in both the incidence and prevalence of peripheral neuropathy is expected in the near future.

Even though the diagnosis of diabetic polyneuropathy can be confidently established with a detailed anamnesis and clinical examination [8,13], electrodiagnostic examination (EDx), including nerve conduction studies (NCS) and needle electromyography, remain as the preferred methods for the detailed assessment of peripheral nerve function and early detection in diabetic patients [8,14]. Potential alternative diagnostic screening methods include skin biopsy and corneal confocal microscopy; however, their validity is yet to be confirmed for implementation in everyday clinical practice [8,11,13]. Currently, EDx serves as the most accessible method for assessing the stage of diabetic neuropathy. When correlated with clinical examination, NCS can confirm the presence of damage to large fiber neurons, whether the involved nerves are sensory or motor, and the severity of the present damage. Repeated NCS can also be used to assess the disease’s progression or response to treatment [8,15].

Existing wearable devices primarily focus on measuring parameters for glycemic control and the detection and management of diabetic angiopathy, leaving a gap in remote and precise early diagnosis of diabetic neuropathy [16].

The aim of this study is to evaluate a telemedicine wearable device with a machine learning (ML) algorithm that can be used as a screening and tracking tool for diabetic neuropathy. Namely, the Moveo platform for Diabetic Neuropathy detection, a proprietary system developed by Divs Neuroinformatics DOO, is assessed against the gold standard of EDx screening.

## 2. Materials and Methods

### 2.1. Protocol Description

This study is characterized as a monocentric, cross-sectional, comparative investigation with two distinct groups: subjects diagnosed with diabetic neuropathy and subjects without diabetic neuropathy. The study was conducted at the Institute of Rheumatology in Belgrade, Serbia in 2022 and received approval from the Ethics Committee of the Institute of Rheumatology (Approval No. 132/30).

The participants in the experimental group were ambulatory patients suffering from diabetes with suspected diabetic neuropathy, while the control group included both healthy volunteers and patients diagnosed with radiculopathy. Inclusion criteria were patients above the age of 18, signed informed consent, no major anatomic abnormalities (amputation, absence of limb, part of limbs, etc.), and no major functional disabilities (paralysis, immobilization, etc.). The exclusion criteria aimed to ensure participant safety and thus disqualified patients with allergies to plastic, anatomical irregularities that complicate proper placement, conditions that could lead to falls (vertigo, Meniere’s disease), and strength abnormalities (myasthenia gravis, multiple sclerosis).

Prior to study enrollment, all subjects were fully informed about the trial and its terms and conditions. Each subject signed a written consent to participate in the study. Prior to enrollment, the subject underwent an initial EDx.

### 2.2. Description of Reference and Investigational Methods

Reference Method: The EDx examination commenced with subjects positioned comfortably on a bed next to the healthcare professional and diagnostic equipment. The electrodes were placed on the limbs, and the examination subsequently started. Measurements of amplitude, latency, and conduction velocity were measured for each nerve studied, including n. medianus, n. ulnaris, n. peroneus, n. tibialis, and n. suralis. During the examination, both motor and sensory fibers were evaluated.

Investigational Method: After the initial exam, the subject took a 15–20 min break to rest while sitting on a chair. A healthcare professional asked if the subject was ready for the next examination; if not, the subject continued resting. If the subject was ready, the Moveo exam could start.

The Moveo exam system consists of 4 sensors that are placed on the back side of the subject’s hands and the upper part of the subject’s feet. These sensors communicate with the Moveo mobile application (compatible with Android and iOS devices), which also additionally facilitates the Moveo exam process. The app instructs the subject to perform a series of 6 tests that are also part of a standard neurological examination. The Moveo platform then makes an estimate regarding the presence and level of diabetic neuropathy and reports it back via the app.

The following exercises were performed during the Moveo examination:

Walk on toes and heels: A person walks, first on his heels, then on his toes, for 10–15 s. The exercise is completed when the subject finishes both types of walks.

Tandem walk: This test is more sensitive when the subject performs the exercise with his eyes closed. H owever, it must be noted that there is a fall risk with this exercise; therefore, this part of the Moveo examination was only performed with the patient’s eyes open. During this exercise, the subject walks with one foot next to the other, aligning his heel next to the toes of the opposite foot. The exercise lasts for 10–15 s. It is performed after the person walks for a specified amount of time.

Romberg test: The subject holds his hands in a straight position (with his arms extended in front of his body). The test is first performed with eyes open, then with eyes closed. The exercise lasts for 10–15 s.

Postural tremor: Tremor occurs when the subject actively maintains a position against the force of gravity (tested on the hands by asking the subject to extend his arms while sitting/standing and maintain that position with and without cognitive distraction for a duration of 10 s). Minimal tremor is expected in healthy people; however, in subjects with neuropathy, this tremor is more expressed.

Finger–nose test: The subject is in a standing position, reaching his nose using his left finger and then his right finger. The test is first performed with eyes open, then with eyes closed.

Heel–knee test: Due to safety reasons, this test is conducted with the subject lying in bed. Subjects are instructed to reach their knee with the heel of the opposite leg, stretching the heel down the leg to the ankle The test is first performed with eyes open, then with eyes closed.

Instructions for patients in the form of application screenshots accompanied with a short explanation can be found in the Supplementary Material file Moveo exercises.

### 2.3. Raw Signal Processing and Features Extraction

**Figure 1:**
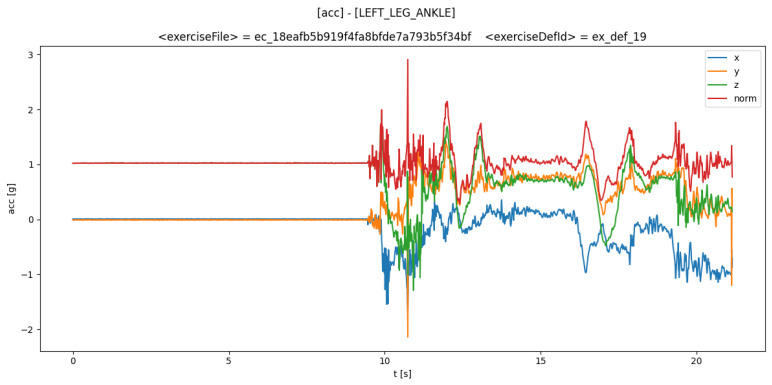
Acceleration signals (*x*, *y*, and *z*) are measured by the sensor, and the *norm* signal is calculated in the cloud during the performance of neurological movement exercises.

Features are calculated are based on raw signals derived from three-axis acceleration and gyroscope measurements. The acceleration signal is composed of three axes (*x*, *y*, and *z*) with each axis carrying some amount of acceleration that the patient produces. A similar approach is applied to the gyroscope signal, which measures angular velocity along the *x*, *y*, and *z* axes. Therefore, the raw signals embody a six degrees of freedom system, incorporation both the accelerometer’s and gyroscope’s *x*, *y*, and *z* axes. An example of the accelerometer signals can be seen in Figure 1.

An additional component of acceleration, attributed to gravity, is also measured using the sensor. Thus, the sensors constantly measure the acceleration component that the user produces plus the component of earth’s gravity (1 G). The first challenge was to extract a useful component that the user produces and reject the gravity. The gyroscope signal (angular velocity) is used to reject gravity by determining the orientation of the gravity vector using angular velocity. Gyroscope measurements involve errors caused by the “gyroscope drift”; thus, we also need to compensate for those errors when determining orientation.

In Figure 1, four distinct signals are shown. Signals annotated with “*x*”, “*y*”, and “*z*” are gathered from the sensor, and the “*norm*” signal is calculated using the Euclidean distance formula:

$$norm=\sqrt{x^{2}+y^{2}+z^{2}}$$

**Figure 2:**
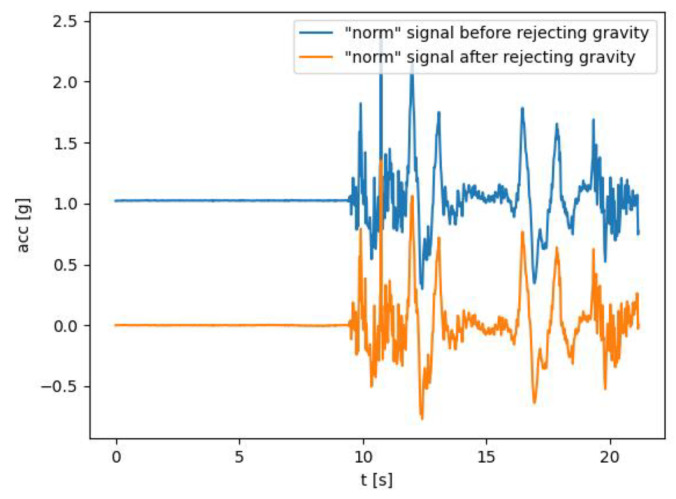
The effects of the method applied for removing the gravity component from the *norm* signal.

Most of the features are calculated using the “*norm*” signal as an input to mitigate issues related to different orientations. Prior to applying the transformation that generates features for the ML model, several preprocessing methods are employed. The initial preprocessing method focuses on filling gaps in the signal resulting from streaming issues. To accomplish this, a second- order polynomial interpolation technique is utilized. After the interpolation phase, many of the features rely on the signal with the gravity component removed. To eliminate the gravity component, a technique based on high-pass filtering is used, specifically employing the “Butterworth’s” high-pass filter with a cut-off frequency of 0.1 Hz. Figure 2 illustrates the behavior of the “*norm*” signal of acceleration before and after gravity removal.

After applying the preprocessing techniques, the signal is ready for final feature extraction. In the following example, one variant of the features used for the development of the ML model is presented.

**Figure 3:**
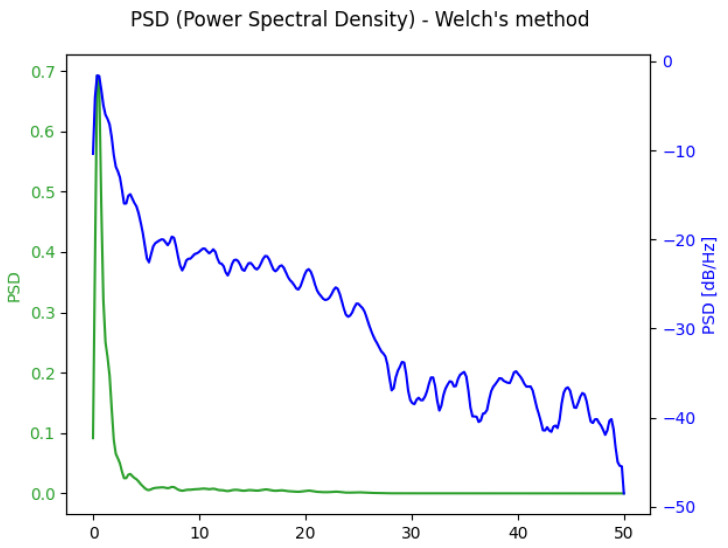
Power spectral density is calculated using Welch’s method applied to the acceleration *norm* signal.

One of the techniques employed to analyze exercise and motion signals in the spectral domain is power spectral density (*PSD*). An example of the *PSD* transformation applied to the signal given above is presented in Figure 3. It displays both the standard *PSD* and the *PSD* in logarithmic format. To calculate the *PSD* using Welch’s method, a window length of 256 is employed. This means that for each calculation of the modified periodogram, 256 signal points are used, with an overlapping factor of 0.5 being selected (Figure 3) [17].

Given that the *PSD* has 256 points, it represents a large input vector for the ML model that should be reduced to avoid overfitting issues. To mitigate this, the final features are the sums of *PSD* points from a specified frequency range, thus reducing the number of points. Some of the cutoff frequencies of interest were 5 Hz and 10 Hz. The equation for the calculation of the final feature based on *PSD* is provided as follows:

$${PSD}_{fx}=\sum_{n=idx(fx)}^{N}PSD[n]$$

where idx(fx) is an index of specified frequency in *PSD* array, and *N* is the length of Periodogram window. In this case, it is 256. The following final features were included:

- *PSD*_5Hz_
- *PSD*_10Hz_
- *PSD*_15Hz_

The extracted features were then used for ML model training. Due to a relatively small amount of data collected during the trial, more complex models (e.g., neural networks) suffered from overfitting. Therefore, several ML models are analyzed to obtain a comparison of which one is the most suitable for this problem, which will be later described in the Results section. Model training and metrics calculations were performed using ML Python library Scikit-learn.

Upon completion of both examinations, participants were asked to complete a questionnaire, which contained 10 questions regarding the two examinations (examined device (Moveo) and comparator (EDx)). No specific scoring has been developed; rather, the questionnaire solely assessed the level of satisfaction regarding each examination.

### 2.4. Statistical Methodology

Results are presented as count (%), means ± standard deviation, or median (25th–75th percentile) depending on the data type and distribution. Groups are compared using parametric (*t*-test) and nonparametric (Chi-square, Mann–Whitney U-test) tests. To assess the correlation between variables, Pearson’s correlation was used. All *p*-values less than 0.05 were considered significant.

This investigation represents a first in human pilot study. Therefore, no prior information related to the effect size was obtained or found; consequently, no sample size has been calculated for this study. The results will be used for further investigations regarding this technology.

The outcome is defined as a binary variable, defined as the presence or absence of diabetic neuropathy.

The machine learning algorithm and all signals were analyzed using Python 3.10.12 and R 4.2.2 (R Core Team (2017). R: A language and environment for statistical computing. R Foundation for Statistical Computing, Vienna, Austria. URL https://www.R-project.org/ accessed on 27 November 2021). For data analysis, data visualization, and ML model training, the following Python packages are used: Pandas (2.2.0), Matplotlib (3.8.12), Scikit-learn (1.2.0), Seaborn (0.3.12), and NumPy (1.26.23).

## 3. Results

The study included 23 participants, including 11 with diabetes and suspected neuropathy (47.8%). Overall, participants were 55.3 ± 12.5 years old, and 8 (34.8%) were males. Eight (72.7%) of the diabetic patients had diabetic neuropathy confirmed via EDx examination. In the diabetic neuropathy group, the mean age was 63.8 ± 8.5 years, which was significantly higher compared to the non-neuropathy group at 50.7 ± 12.0 years (*p* = 0.012). The diabetic neuropathy group had 3 (37.5%) male participants, which is very similar to the non-neuropathy group with 5 males (33.3%) (*p* = 1.000).

All Moveo features were analyzed; however, only the most important features that can be used to differentiate neuropathy patients versus non-neuropathy patients are presented in Table 1. Additionally, other features that were expected to be of importance are also presented, even though these features revealed no statistical significance or clinical differences.

In the heel–toe walk test, two features were significant, while in the tandem walk test, no significant features were obtained. The level of significance of feature 5 is near the conventional level that was considered when the modeling was performed. The heel and knee test also revealed two significant features. In the arms tests, the Romberg test revealed only one significant feature. Regarding postural tremor, only one feature was determined to be at the level of statistical significance (*p* < 0.05); however, one more feature was close to the conventional level of significance that was considered during the modeling. In the finger–nose test, two significant features were obtained.

To present the individual characteristics of the features, the correlation matrix with the most important Moveo features and EMNG parameters are presented in Table 2. The most important features are those that have the highest Pearson’s correlation coefficient with the output binary variable (no DN/DN). Then, those features are analyzed to determine the correlation with EMNG parameters. As shown, the coefficients vary from insignificant to above 0.6. Every Moveo feature had at least one correlation with an EMNG feature with a correlation coefficient above 0.4, while half of the features revealed correlations with values above 0.5. Several features revealed correlation coefficients above 0.6.

Using the most important features, several ML models for binary classification were developed. To avoid overfitting, the one-by-one feature elimination approach was applied based on *p*-value as well as the correlation among features in order to not pick two features with high correlation between each other because those features bring the same information. Also, to prevent overfitting, dataset synthesis based on the Kernel Density method [18] was applied. A Gaussian kernel was employed for this purpose that enabled the generation of 200 negative and 200 positive samples from the initial 23 samples. Consequently, the final dataset that was used for training had 423 samples.

For the sake of results comparison, three ML models were used in the analysis: support vector machine (SVM), logistic regression, and decision tree (DT). During the training for each model, the Grid Search algorithm was applied with the corresponding parameters grid, to build an ML model with optimal parameters.

For each Moveo exercise, a unique ML model was trained, and the final results were calculated using the following formula:

$${output}_{aggregated}=\frac{1}{6}\sum_{i=0}^{6}{ExerciseModel}_{i}(X_{i})$$

where $ExerciseModel_{i}$ represents the ML model for *i*-th Moveo exercise and can be one of the three analyzed ML models, and $X_{i}$ is an input vector that contains selected features for the corresponding exercise.

**Figure 4:**
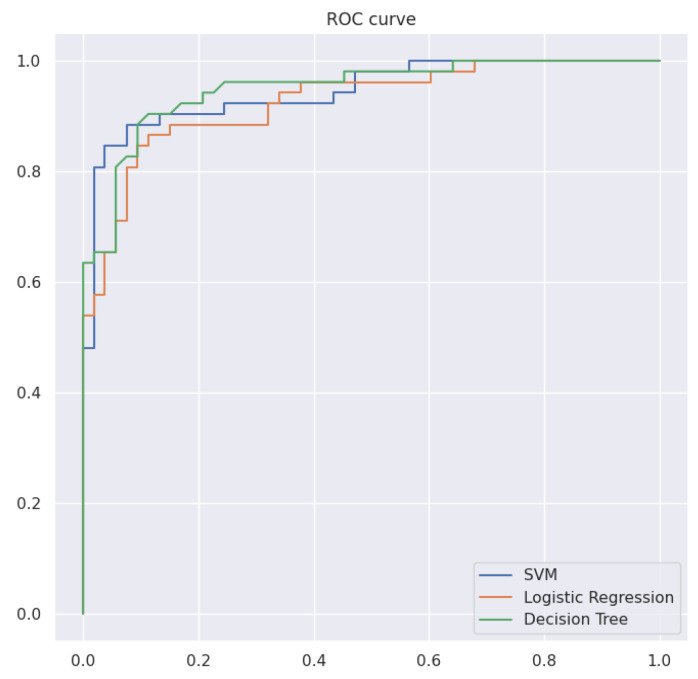
ROC curve across ML models.

Table 3 and Figure 4 show the comparison of ML model metrics. The SVM model showed the best result in comparison with logistic regression and DT because of its flexibility in terms of the kernel. In this case, the SVM model for the “heel–knee” exercise can use a linear kernel if classes are more linear separable for “heel–knee” features, while the SVM model for “Romberg’s test” exercises can use a radial basis or polynomial kernel if classes are non-linear separable for “Romberg’s test” features. The dataset is split to train and test at a ratio of 70:30. Results that are shown are generated from the test dataset. Performances in Table 3 and Figure 4 are obtained by averaging output from the ML model. The aggregated output is described in the above equation and compared with the true output. The aggregated model based on SVM achieved a sensitivity of 0.78 and a specificity of 0.87.

The distribution of patients regarding the answers of the user experience questionnaire are presented in Table 4.

The user experience questionnaire revealed significant differences between the EMNG and Moveo examinations. The differences are obvious and extreme with regard to unpleasant examination, pain, fear, and duration. The Moveo examination was deemed superior in all aspects compared to EMNG. The last question was “if the patient can choose which examination, he/she would choose?”. The majority of patients [21 patients (91.3%)] would choose Moveo, while 2 patients (8.7%) would choose any technology.

## 4. Discussion

With the increase in the incidence of diabetes, an increase in the incidence of diabetic polyneuropathy has also been observed. Thus, it is especially important that people suffering from diabetes, a multi-organ disease, are monitored by a multidisciplinary team of physician specialists. In order to adequately monitor and treat diabetic neuropathy, the patient should be referred to a neurologist at least once a year after being evaluated by their endocrinologist [9]. During the subsequent examination, the neurologist may require further diagnostics, such as EDx. In the context of the increasing prevalence of diabetes, the need for a sufficient number of specialists, EDx devices, and examination appointments are essential. There has been a significant upward trend regarding the usage of modern technologies in medicine, thus opening the doors for potential telemedicine solutions.

Moveo is a novel device that aims to reduce EDx wait lists as well the workload of healthcare staff. Self-examination is already common practice in some medical fields, such as endocrinology and cardiology, but Moveo has the potential to open up this practice to an additional field, namely, neurology. Moveo is a device that comprises an aluminum case and silicon band with a sensor inside the case that records movement during the six exercises (see https://www.divstechnology.com/, accessed on 15 December 2021, for more information). The signal is transferred to the mobile device (phone, tablet) using low- energy Bluetooth technology and processed using the pre-defined machine learning algorithm.

Telemedicine-only examinations were introduced as a novelty, where the patient, according to the given instructions, performs the examination on the basis of his answers to certain questions, which are then sent back to the physician [19]. Moveo exercises are explained using cartoon-style recorded instructions so that the user has explicit instructions on how to perform the exercise and how to enter the data, if needed.

The primary goal of monitoring diabetic patients is the adequate regulation of glycemia and its maintenance within the target values based on a hygienic diet regimen and therapy. Glycemic control is directly correlated with the occurrence of chronic microvascular complications of diabetes [16]. The Moveo device tracks the consequences of a non-regulated trend rather than the glucoregulation trend; however, it is obvious that the patient’s score correlates with the glucoregulation status and behavioral patterns that influences the results of the diabetic neuropathy score. In newly diagnosed patients, it can be used as a screening tool for the first signs of diabetic neuropathy. In more advanced stages and in patients who already present with neuropathy, it could be used as a tool for neuropathy trend evaluation and as an alarm if the disease has progressed more than expected.

Currently, screening for diabetic neuropathy is based on symptoms and signs and therefore is limited to a clinical examination carried out by a neurologist at least once a year. The neurological examination itself is long and detailed, consisting of inspecting the sensory modalities of the patient’s skin, usually temperature, light touch, and vibration, as well as trophic and muscle strength, with a reflex check [9,15,20]. Moveo offers a completely different approach since it dynamically examines the movement disorders as a result of diabetic neuropathy. Compared to a static examination like EDx, Moveo tests the variation in movement as a consequence of a nerve conduction disorder. With regard to the above-mentioned concept stated in our manuscript, several other authors have proposed different approaches to detecting and tracking diabetic neuropathy [21]. Some of them hypothesized that with diabetes (and peripheral neuropathy) progression and other confounding factors (such as cognitive impairment), patients are more prone to changes in the gait pattern and balance alterations, resulting in an increased risk of falling [22,23,24,25,26,27,28,29], with another approach consisting of a camera capturing the motion [30,31,32]. A few articles bring up the idea of utilizing ML on collected NCS data in terms of neuropathy prediction [33]. Other studies investigated the usage of thermal imaging [34,35], ultrasound [36], vibration [37], plantar pressure [38,39,40,41], temperature [42], and corneal nerve evaluation [43]. However, none of these studies provide a comparison of a wearable sensor-based approach with a “gold standard” NCS method for neuropathy confirmation to evaluate the true accuracy of the proposed models. Moreover, in our study, we used exercises that were a potential surrogate for a neurological examination.

In diabetics, peripheral nerve damage occurs most often in the lower extremities, symmetrically, affecting first the thin sensory fibers (A delta, B and C fibers) that are more susceptible to metabolic abnormalities. As the disease progresses, the large motor fibers are also affected [22]. After clinical examination, if the physician suspects diabetic peripheral neuropathy, NCS and needle electromyography (EMG) are used because these methods show a high degree of sensitivity in detecting damage to large peripheral nerve fibers. Both methods are usually included under the name of electroneuromyography (EMNG) or more precisely EDx [15,20]. Sensory and motor nerve conduction examination is performed using electric stimulation and recording over distal lower limb segments and then upper limb segments [15]. Moveo has tests for both legs and arms. A part of a typical neurological examination is performed with sensors on the arms and legs. The movement is being recorded, and processed signals are translated to values used for score calculation. It is expected that the leg examination is more accurate for neuropathy diagnosis. However, if the results are positive for the arms, it is more likely that there is an advanced stage of neuropathy present due to proximal nerve disorders, which occur later.

In the first test assignment (walking on the toes and heels), the analysis revealed that the features that test the dynamics of movement were noted as the most important features. Sensors placed on the foot in the heel– toe walk and features that were derived from those sensors showed that walking depended on foot strength, which can be reduced in diabetic neuropathy.

In the other five exercises, features that compare movement when the subject has his/her eyes open and eyes closed are considered to be the most important for DMN discrimination. Since deep postural sensibility can be damaged in diabetic neuropathy, when the subject closes his/her eyes, accurate movement in dynamic exercises will be impaired. Similarly, their capacity to maintain a stable position in static exercises will also be impaired, underscoring the importance of these features.

Targeted measures are the amplitude of the evoked response (APA) and parameters that reflect sensory and motor nerve conduction velocity (NCV), which include distal latency (DL), F-wave latency, and conduction velocity (CV). Abnormalities in these parameters are specific markers for peripheral nerve disease [9,15]. Moveo results correlate significantly with the EDx findings, and there are a significant number of highly correlated parameters that could be interpreted as surrogates for EDx. Future studies with larger samples will aim to further analyze this in order to assess the compatibility of Moveo and EDx. However, based on the results of the pilot study, this technology could be considered very promising and possibly used in the near future. Using a ML algorithm with chosen features, excellent classification power is observed. Future studies will aim to prove non-inferiority of Moveo compared to gold standard diagnostic tools for diabetic neuropathy diagnostics. Moveo’s reproducibility result is still questionable due to insufficient testing of the device. In the future, the aim is to obtain an algorithm that will provide stable results without outliers and noise. The increased sample size and number of patients with DN will allow for better estimation of diagnostic accuracy measurements (Sn, Sp, PPV, NPV, and total accuracy).

From an economic point of view, it must be considered that the effects and, in some cases, the benefits of such examinations are financially worthwhile. Based on savings on transportation, space, and time, the difference in cost can be even 10 times cheaper [44]. Using the Moveo device, the patient can perform the exercises and record the report to the device. The average costs of an EDx vary from country to country. The average price in the USA is between $200 and $1200 depending on state, practice, etc. Comparing the prices of specialist examination (general examination of neurologist or physiatrist), the EMNG examination price is 2–4 times higher. Contrarily, Moveo is a very cost-effective tool. The user can do it at home by themselves, and the results can be used to alarm the physician of any non-favorable trends. The device costs are less than the average cost of one EDx. The adoption of frequent self-examination practices among diabetic patients coupled with increased awareness of the potential consequences of an unhealthy lifestyle present significant benefits for healthcare providers as well. Over time, this can lead to a reduction in the demand for physician services, thereby alleviating the overall workload on healthcare providers, ultimately streamlining healthcare delivery.

As a part of the evaluation of telemedicine, the perspectives of both patients and healthcare workers should be considered. Patients expressed greater satisfaction with telemedicine compared to conventional healthcare given that the entire process takes place in the comfort of their homes, which increases patient adherence [45,46,47]. Health workers have shown a great desire and willingness to adapt their skills to telemedicine to provide healthcare [45], with the statement that such working conditions are more optimal [47].

Although the application of ML in disease diagnosis is already an ever-expanding field of research, its development and application in diabetic peripheral neuropathy diagnosis is still very uncommon. This is notable given the critical need for precise, early diagnosis and classification of diabetic peripheral neuropathy severity to facilitate timely and appropriate treatment interventions. Existing literature reveals the implementation of different conventional ML-based classifiers that are able to classify diabetic neuropathy severity levels using scores that are based on a patient-filled questionnaire, clinical parameters, and NCS data obtained through physical examination and NCS assessment [33,48,49]. With regard to ML applications in electrodiagnostic assessments of diabetic peripheral neuropathy and further staging, research was conducted aimed at optimization of the ML algorithm for observation of the biomechanical changes in muscle electromyography (EMG) and ground reaction forces (GRF) through signal preprocessing with strategic feature extraction from the EMG and GRF signals, offering a potential solution for the diagnosis and stratification of patients with neuropathy [50].

## 5. Conclusions

Moveo represents an accurate and promising technology for the screening, diagnosis, and follow-up of diabetic neuropathy. It represents a satisfactory replacement method for EDx, which patients generally dislike and consider uncomfortable and painful. Several limitations are worth mentioning. First, EDx is a gold standard for the diagnosis of diabetic neuropathy but only when larger fibers are affected. In the early stages of the disease, small fibers can be affected, and the patient could be labelled as healthy (false negative). However, when small fibers are affected (such as A gamma and delta, B, and C fibers), patients could also exhibit different sensations such as pain and discrete motor function alterations that can influence movement and be detected by sensors. Further studies with a larger sample size are needed to evaluate the true accuracy of the technology with regard to large-fiber diabetic neuropathy; however, for small-fiber diabetic neuropathy, different tests should be used as a gold standard for comparison.

## 6. Patents

DIVS Neuroinformatics D.O.O. has a patent submission on Moveo as a system for determining levels of peripheral nerve impairment that has been submitted to Intellectual Property Office of Serbia under number P-2022/0985.

## Figures and Tables

**Table 1:** Moveo features in diabetic neuropathy and non-neuropathy participants.

		DM Neuropathy		*p* Value
		No (n = 15)	Yes (n = 8)	
Legs	Heel–toe walk			
	Feature 1	0.011 ± 0.005	0.005 ± 0.003	0.013
	Feature 2	1.481 ± 0.369	1.402 ± 0.334	0.620
	Feature 3	1.459 ± 0.300	1.418 ± 0.363	0.778
	Feature 4	0.107 ± 0.048	0.056 ± 0.031	0.014
	Tandem walk			
	Feature 5	1.481 ± 0.206	1.327 ± 0.135	0.072
	Feature 6	1.307 ± 0.094	1.230 ± 0.138	0.126
	Heel–knee			
	Feature 7	0.036 ± 0.029	0.012 ± 0.009	0.008
	Feature 8	0.068 ± 0.104	0.021 ± 0.014	0.107
	Feature 9	0.052 ± 0.072	0.034 ± 0.059	0.557
	Feature 10	0.695 ± 0.304	0.442 ± 0.173	0.042
Arms	Romberg test			
	Feature 11 × 10^3^	0.164 ± 0.131	0.339 ± 0.265	0.044
	Feature 12 × 10^5^	0.109 ± 0.156	0.298 ± 0.282	0.050
	Feature 13	0.628 ± 0.140	0.542 ± 0.169	0.206
	Feature 14	0.604 ± 0.173	0.505 ± 0.190	0.218
	Postural tremor			
	Feature 15	0.017 ± 0.011	0.011 ± 0.012	0.236
	Feature 16	0.020 ± 0.013	0.013 ± 0.008	0.184
	Feature 17	1.044 ± 0.487	0.707 ± 0.244	0.082
	Feature 18	1.281 ± 0.788	0.839 ± 0.370	0.151
	Feature 19	0.638 ± 0.154	0.569 ± 0.159	0.326
	Feature 20	0.597 ± 0.172	0.564 ± 0.164	0.664
	Finger–nose test			
	Feature 21	1.006 ± 0.025	0.960 ± 0.062	0.018
	Feature 22	0.003 ± 0.012	−0.021 ± 0.033	0.017
	Feature 23	0.598 ± 0.195	0.548 ± 0.156	0.537
	Feature 24	0.606 ± 0.202	0.616 ± 0.218	0.911

Due to patent protection, only feature numbering is presented instead of feature name and characteristics.

**Table 2:** Correlation matrix between most important features and EMNG parameters.

	DMN	Feat. 1	Feat. 4	Feat. 5	Feat. 6	Feat. 7	Feat. 10	Feat. 11	Feat. 12	Feat. 16	Feat. 17	Feat. 21	Feat. 22
Motor													
Arm													
Amp LM ra	−0.109	0.255	0.268	0.147	0.160			0.379	0.185	0.264	0.255		
Lat LM pr	0.591		−0.235	−0.181	−0.120	0.652	0.387		−0.214	−0.372	−0.329	−0.141	
CV LM pr	−0.872	0.650	0.596	0.593	0.443	−0.200	−0.199	0.329	0.182	0.426	0.437	0.526	0.413
Lat LU ra	0.213	−0.202	−0.259	−0.280	−0.187	0.171			0.245			−0.168	0.131
AMP LU ra	−0.139			0.240	0.312	−0.205	0.457	0.261	0.217	0.341	0.288		−0.111
Lat LU pr		0.336			0.261	0.143		0.263	0.161	0.149	0.182	0.256	0.408
Amp LU pr	−0.128			0.243	0.277	−0.136	0.375	0.359	0.332	0.332	0.302		
CV LU pr		−0.363		−0.444	−0.496	−0.171		−0.174				−0.167	−0.221
Leg													
Lat RT sa	0.584	−0.162	−0.155	−0.335	−0.344	0.417	0.458		−0.119			−0.189	
Amp RT sa	−0.489	0.291	0.429	0.309	0.160	−0.650	−0.337	0.335	0.167	0.165	0.144	0.247	0.448
Lat LT sa	0.456	−0.104	−0.395	−0.227	0.116	0.484	0.505			0.281	0.296		
Amp LT sa	−0.233		0.101		0.184	−0.506		0.143	0.161	0.128		0.116	
Lat RP se	0.260				−0.145	0.508	0.254	0.199		−0.201	−0.140		
Amp RP se	−0.430	0.515	0.283	0.211	0.250	−0.692	−0.479		−0.193	0.373	0.339	0.496	0.193
Lat RP ps	0.303	0.120	0.149		−0.127	0.350	0.236	0.285					0.202
Amp RP ps	−0.434	0.466	0.194	0.245	0.345	−0.663	−0.503		−0.132	0.396	0.359	0.477	0.166
CV RP ps	−0.465	0.190	0.214	0.333	0.212	−0.577	−0.436	0.173				0.250	
Lat LP se	0.281		0.115	0.145	0.152	0.323		0.219					0.127
Amp LP se	−0.292	0.505	0.287	0.327	0.357	−0.643	−0.227			0.362	0.330	0.716	0.448
Lat LP ps	0.371					0.622	0.300						0.200
Amp LP ps	−0.348	0.536	0.351	0.292	0.341	−0.640	−0.282			0.402	0.380	0.704	0.493
CV LP ps	−0.490		0.149	0.112		−0.657	−0.283			0.203	0.154	0.255	
Sensory													
Arm													
Lat LM rp	0.499	−0.339	−0.435	−0.233	−0.171	0.722	0.282	−0.144		−0.583	−0.544	−0.352	−0.131
Amp LM rp	−0.351	0.445	0.432	0.331	0.281	−0.589	−0.260		−0.139	0.402	0.388	0.451	0.546
CV LM rp	−0.559	0.345	0.454	0.201	0.176	−0.658	−0.345	0.124	0.179	0.429	0.405	0.421	0.273
Lat LU rp	0.314		−0.216	−0.145		0.351				−0.125		−0.115	
Amp LU rp	−0.413	0.418	0.474	0.411		−0.498	−0.157		−0.105	0.294	0.283	0.391	0.402
CV LU rp	−0.374	0.140	0.228			−0.402		−0.139		0.293	0.261	0.298	0.247
Leg													
Lat RS ps	0.506	−0.230	−0.222	−0.174		0.380	0.458		0.208	−0.411	−0.412		
Amp RS ps	−0.539	0.391	0.332	0.515	0.194	−0.344	−0.353	0.209		0.278	0.285	0.341	0.528
CV RS ps	−0.674	0.320	0.353	0.226		−0.446	−0.596	0.197	0.126	0.312	0.311	0.264	0.148
Lat LS ps	0.693	−0.300	−0.475	−0.133	0.113	0.335	0.253	−0.248	−0.116	−0.188	−0.168		−0.168
Amp LS ps	−0.694	0.285	0.432	0.243		−0.455	−0.497	0.252	0.289	0.237	0.236	0.193	0.436
CV LS ps	−0.731	0.400	0.500	0.218		−0.400	−0.468	0.297	0.210	0.217	0.212	0.265	0.215

Feat.—feature; Motor—motor nerves; Sensory—sensory nerves; DMN—diabetic neuropathy; Arm/Leg—examined limb; Lat—latency; Amp—amplitude; CV—conduction velocity. LM—left medianus; LU—left ulnaris; RT—right tibialis; LT—left tibialis; RP—right peroneus; LP—right peroneus; RS—right suralis; LS—left suralis. ra, pr, sa, se, ps, rp, ps—location of second electrode for EMNG measurement. light gray—correlation coefficient between 0.4 and 0.6; dark grey—correlation coefficient above 0.6; correlation coefficients below 0.100 are presented as empty fields.

**Table 3:** Metrics comparison across different ML models.

	Precision	Recall	f1-Score	Supprt
SVM-no DN	0.759259	0.872340	0.811881	47.0
SVM-DN	0.884615	0.779661	0.828829	59.0
Logistic Regression-no DN	0.592593	0.761905	0.666667	42.0
Logistic Regression-DN	0.807692	0.656250	0.724138	64.0
Decision Tree-no DN	0.629630	0.809524	0.708333	42.0
Decision Tree-DN	0.846154	0.687500	0.758621	64.0

**Table 4:** User experience questionnaire by examination.

	Examination		*p* Value ^a^
	EMNG	Moveo	
Unpleasant			
No	1 (4.3%)	21 (91.3%)	<0.001
Mild	7 (30.4%)	1 (4.3%)	
Moderate	11 (47.8%)	1 (4.3%)	
Severe	4 (17.4%)	0	
Pain			
No	0	22 (95.7%)	<0.001
Mild	8 (34.8%)	1 (4.3%)	
Moderate	11 (47.8%)	0	
Severe	4 (17.4%)	0	
Fear			
No	10 (43.5%)	22 (95.7%)	<0.001
Mild	5 (21.7%)	1 (4.3%)	
Moderate	5 (21.7%)	0	
Severe	3 (13.0%)	0	
Duration			
As it should be	5 (21.7%)	22 (95.7%)	<0.001
Little longer	12 (52.2%)	1 (4.3%)	
Much longer	6 (26.1%)	0	

^a^ Pearson Chi-square test.

## Data Availability

Moveo as a system has patent submission, and the algorithm is owned by DIVS Neuroinformatics D.O.O. Data used in this study are not available because it might be used against the interest of DIVS Neuroinformatics D.O.O. Therefore, in this phase of research, the data will not be publicly available.

## Supplementary Materials

The following supporting information can be downloaded at: https://www.mdpi.com/article/10.3390/bios14040166/s1, Moveo exercises.pdf with exercise descriptions and application screenshots.
