# Supplementary material for: Wearable Movement Exploration Device with Machine Learning Algorithm for Screening and Tracking Diabetic Neuropathy—A Cross-Sectional, Diagnostic, Comparative Study
Source: Biosensors (Basel). 2024 Mar 29;14(4):166. doi: 10.3390/bios14040166 (PMC11047826; doi:10.3390/bios14040166)

Moveo app instructions for users

The following text and pictures are from Moveo application for each of six exercises used in the examination.

User places the sensors on the hand and feet and starts the application.

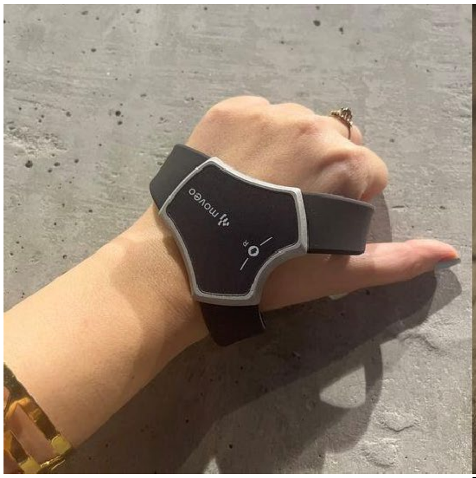

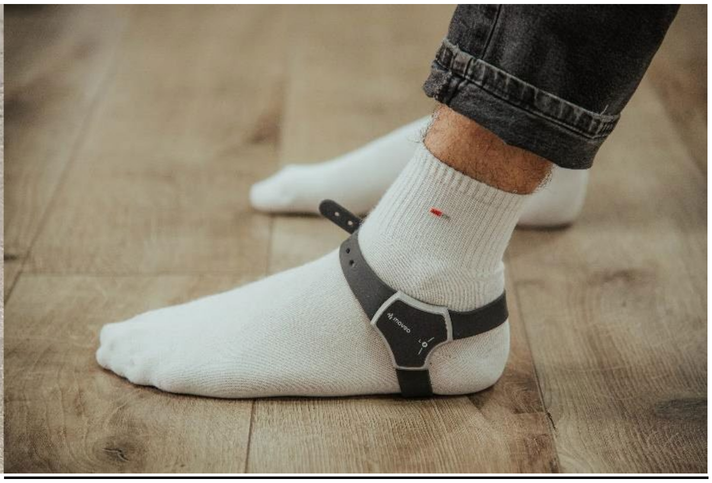

The application guides user through each exercise using video and sound.

HAND exercises

Two exercises are in sitting position (finger-nose and postural tremor) while Romberg test can be performed in standing, and if the patient is not stabile, in sitting position.

<u>Finger – Nose</u>

Patient is in sitting position with open arms. With his index finger he needs to touch the apex of his nose first with open, and then closed eyes.

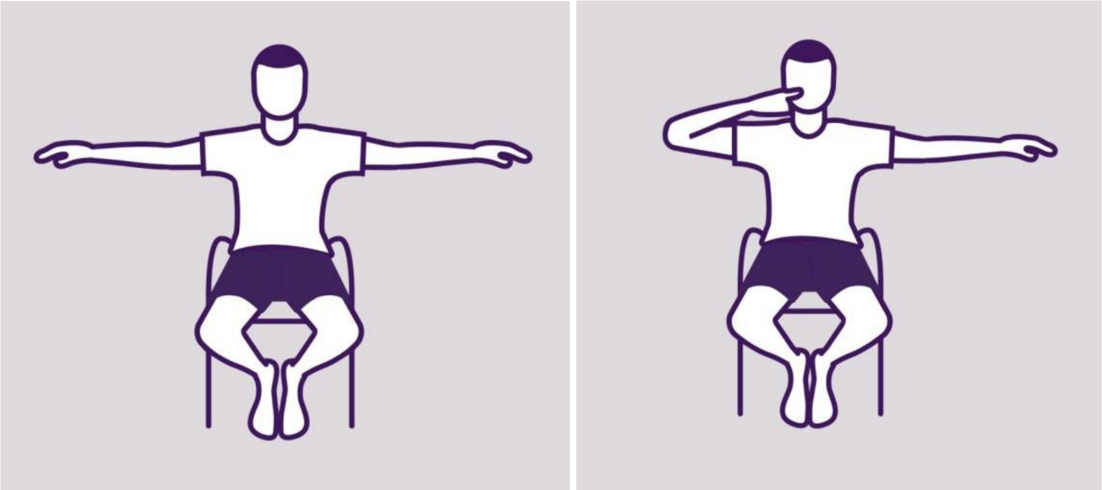

Romberg test

Patient is standing with arms next to the body and after the sound rises arms in straight position. After 10 seconds, after the sound, the patient closes eyes and continue to stand for next 10 seconds. After the sound the exercise is over, the patient opens his eyes and put arms in starting position.

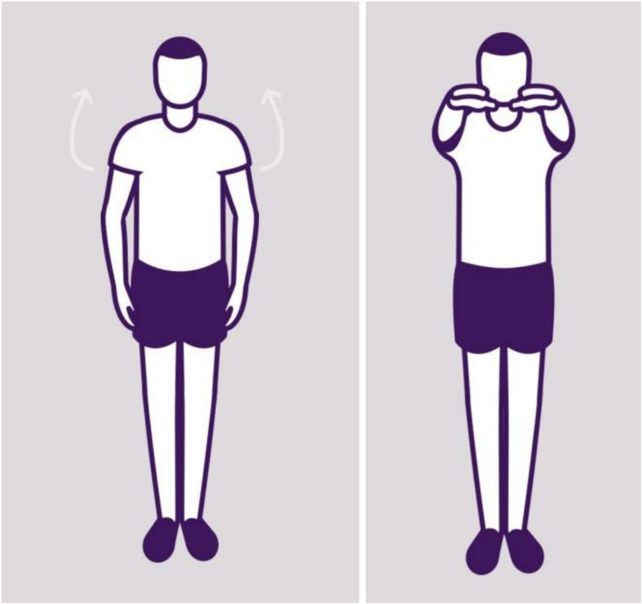

Postural tremor

Patient is in sitting position. After the sound the patients rises hand in straight possition. Examiner ask a patient to do some distractive action (count days or month backward etc.). When the patient is finished, the exercise is over, patient put his arm in starting position and recording stops.

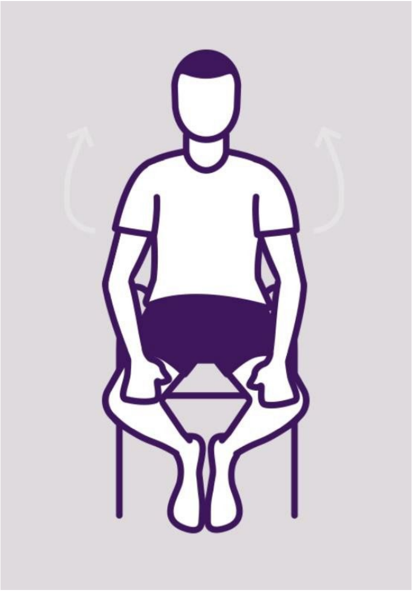
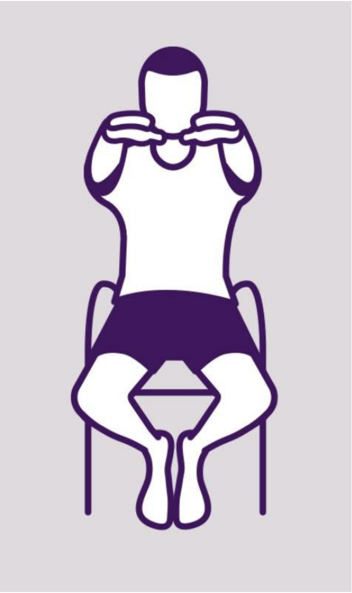

LEG exercises

Two excercises are in standing position (walk on heals and toes, tandem walk) and one is for safety reason in sitting or lying position (heel-knee).

<u>Heel – Knee</u>

Patient is lying position. After the sound, with the subject in sitting position, they are instructed to place the heel of one foot onto the knee of the other leg and then slide the heel down the shin from the knee to the ankle and back up to the knee.

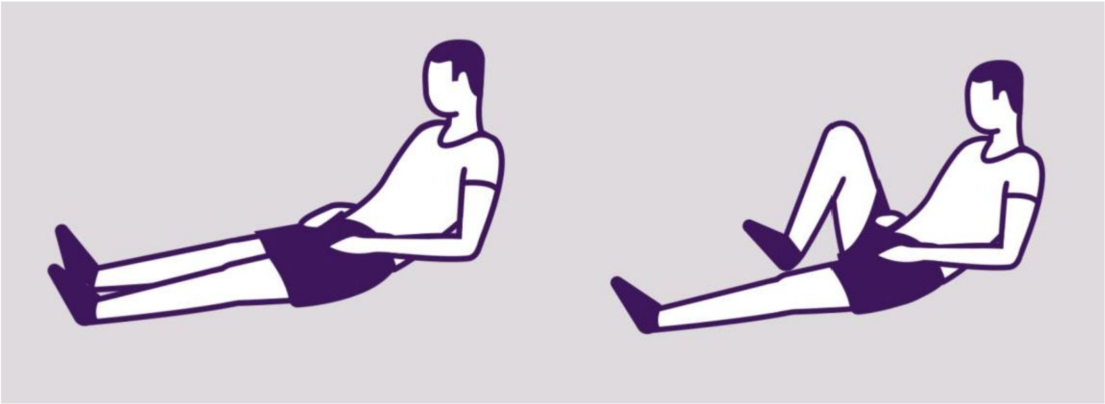

Walk on the heals and toes

User walks on the heels for 4 seconds and then after the beep sound, walks on the toes for 4 seconds (approximately 8 meters).

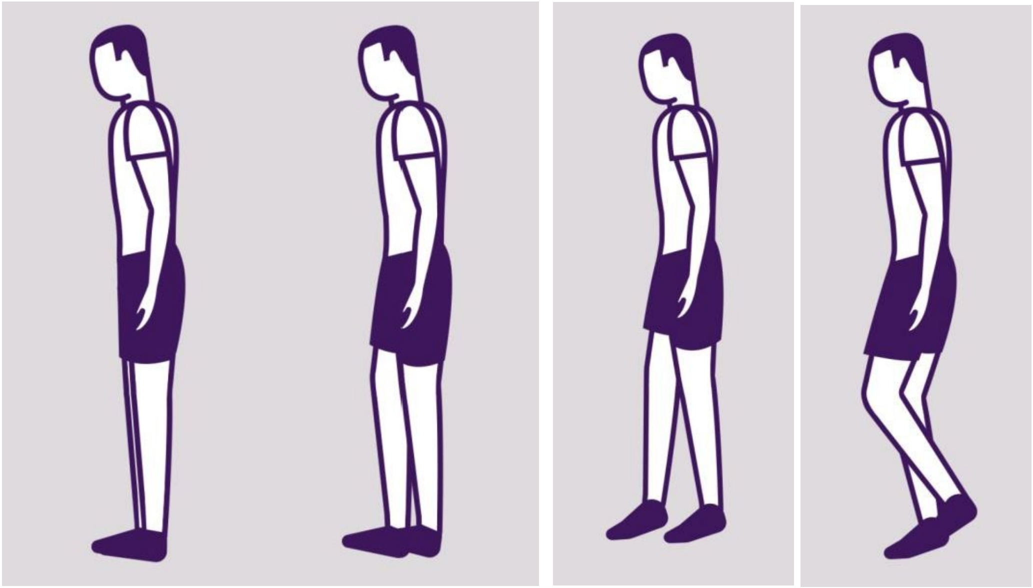

Tandem walk

After the sound signal user starts walking in a straight line with one foot immediately in front of the other (heel to toe), arms down by their side aproximately 8 meters.

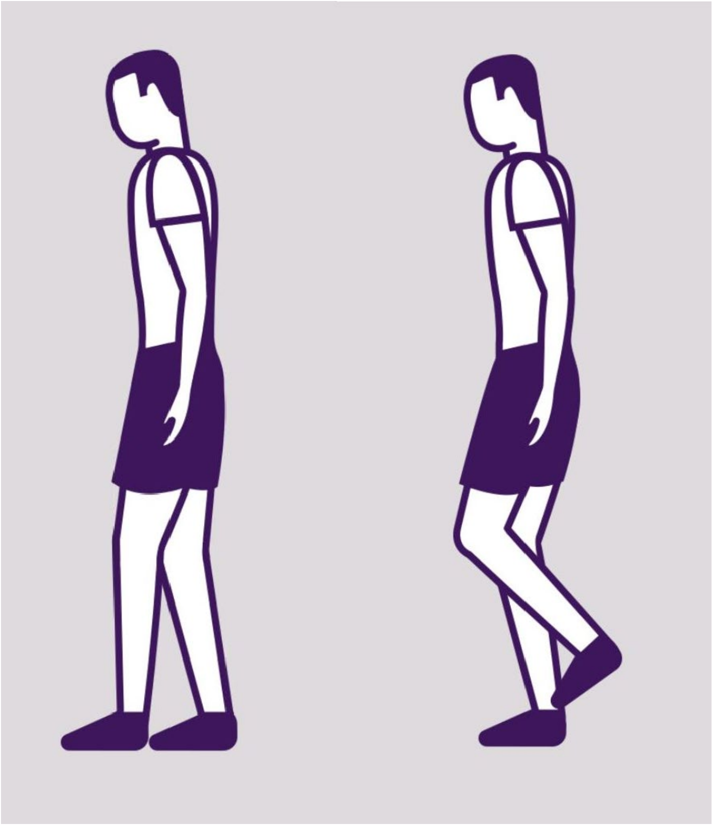

<u>FINAL REPORT</u>

Using the machine learning algorithm, Moveo calculates diabetic neuropathy score and present the results to the user. The results are explained using text and colors which encourages or alarms him, depending on the score.

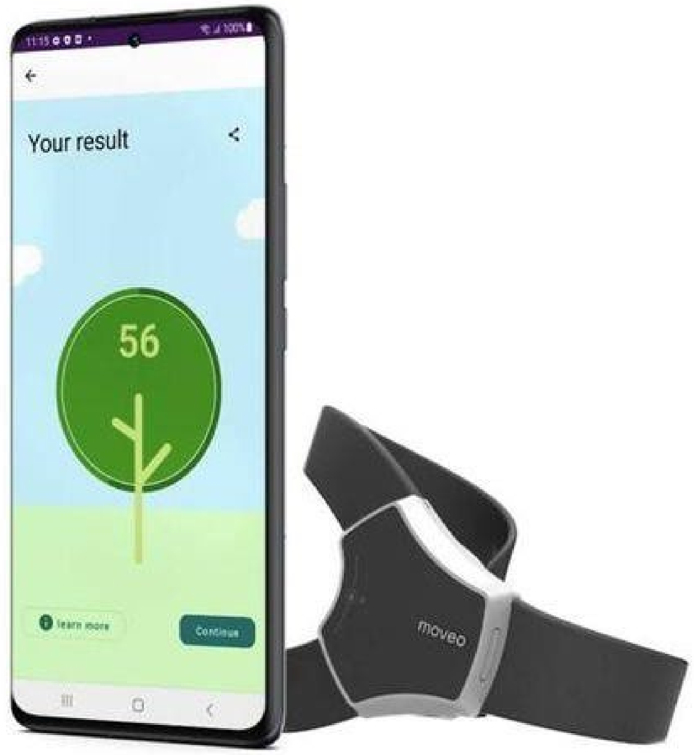

Supplement: Supplementary file 1 [file biosensors-14-00166-s001.zip › biosensors-2898227-supplementary.pdf]
